# Crassolide Induces G2/M Cell Cycle Arrest, Apoptosis, and Autophagy in Human Lung Cancer Cells via ROS-Mediated ER Stress Pathways

**DOI:** 10.3390/ijms23105624

**Published:** 2022-05-17

**Authors:** Kuan-Ming Lai, Jou-Hsuan Wang, Shih-Chao Lin, Ya Wen, Chao-Liang Wu, Jui-Hsin Su, Chien-Chin Chen, Chi-Chien Lin

**Affiliations:** 1 Program in Translational Medicine, National Chung Hsing University, Taichung 402, Taiwan; 143830@cch.org.tw; 2 Hemato-Oncology Division Department of Internal Medicine, Changhua Christian Hospital, Changhua 500, Taiwan; 3 Institute of Biomedical Science, The iEGG and Animal Biotechnology Center, National Chung-Hsing University, Taichung 402, Taiwan; doris8569@smail.nchu.edu.tw; 4 Bachelor’s Degree Program in Marine Biotechnology, College of Life Sciences, National Taiwan Ocean University, Keelung 202, Taiwan; sclin@mail.ntou.edu.tw; 5 Department of Physiology and Pharmacology, Karolinska Institutet, SE-171 77 Stockholm, Sweden; ya.wen@ki.se; 6 Department of Medical Research, Ditmanson Medical Foundation Chia-Yi Christian Hospital, Chiayi 600, Taiwan; wumolbio@mail.ncku.edu.tw; 7 National Museum of Marine Biology and Aquarium, Pingtung 944, Taiwan; x2219@nmmba.gov.tw; 8 Department of Pathology, Ditmanson Medical Foundation Chia-Yi Christian Hospital, Chiayi 600, Taiwan; 9 Department of Cosmetic Science, Chia Nan University of Pharmacy and Science, Tainan 717, Taiwan; 10 Department of Medical Research, China Medical University Hospital, Taichung 404, Taiwan; 11 Department of Medical Research, Taichung Veterans General Hospital, Taichung 407, Taiwan; 12 Department of Pharmacology, College of Medicine, Kaohsiung Medical University, Kaohsiung 807, Taiwan; 13 Department of Biotechnology, Asia University, Taichung 413, Taiwan

**Keywords:** autophagy, apoptosis, cell cycle, crassolide, soft coral, ER stress, lung cancer, reactive oxygen species

## Abstract

Crassolide, a cembranoid diterpene extracted from the soft coral *Lobophytum crissum*, has been proven to possess antioxidant and immunomodulatory properties. In the present study, we assessed the anticancer effects of crassolide on human H460 non-small-cell lung cancer (NSCLC) cells. We found that crassolide exerted cytotoxic effects on H460 cancer cells in vitro, inducing G2/M phase arrest and apoptosis. In addition, in H460 cells exposed to crassolide, the expression of the autophagy-related proteins LC3-II and beclin was increased, while the expression of p62 was decreased. Moreover, inhibiting autophagy with chloroquine (CQ) suppressed the crassolide-induced G2/M arrest and apoptosis of H460 cells. Moreover, we also found that crassolide induced endoplasmic reticulum (ER) stress in lung cancer cells by increasing the expression of ER stress marker proteins and that the crassolide-induced G2/M arrest, apoptosis, and autophagy were markedly attenuated by the ER stress inhibitor 4-phenylbutyric acid (4-PBA). Furthermore, we found that crassolide promoted reactive oxygen species (ROS) production by H460 cells and that the ROS inhibitor N-acetylcysteine (NAC) decreased the crassolide-induced ER stress, G2/M arrest, apoptosis, and autophagy. In conclusion, our findings show that crassolide inhibits NSCLC cell malignant biological behaviors for the first time, suggesting that this effect may be mechanistically achieved by inducing G2/M arrest, apoptosis, and autophagy through ROS accumulation, which activates the ER stress pathway. As a result of our findings, we now have a better understanding of the molecular mechanism underlying the anticancer effect of crassolide, and we believe crassolide might be a candidate for targeted cancer therapy.

## 1. Introduction

Lung cancer is one of the most common cancers, and it is responsible for the deaths of 1.4 million people each year. NSCLC (non-small-cell lung cancer) is a kind of lung cancer that accounts for approximately 80–85% of all lung cancer cases [1,2]. The preferred treatment of NSCLC is surgical excision at an early stage and chemotherapy, radiation, targeted therapy, and other forms of sophisticated therapy at a later stage; the 5-year survival rate of patients with NSCLC is approximately 15% [3]. The long-term benefits of these targeted medicines are limited by their significant side effects, substantial cytotoxicity, and therapeutic resistance [3]. The search for innovative alternatives for NSCLC therapy with fewer side effects continues.

Cembranolides are diterpenoids of the cembrane class that have been organically isolated from marine creatures [4]. Cembranolides are characterized by a fourteen-membered carbocyclic ring skeleton with a five-, six-, seven-, or eight-membered lactone ring. Cembranolides have received substantial attention in recent years because of their distinct structural properties and biological activities [5]. In addition, the cembrane derivatives of soft coral *Lobophytum* species have been demonstrated to have antivirus, immunostimulatory, anti-inflammatory, anticancer, and antibacterial activities [6,7,8,9,10].

Crassolide is one of the most prevalent cembranoid diterpenes isolated from corals, such as *Sarcophyton crassocaule* and *Lobophytum crassum*; it has a distinctive structural scaffold, and its bioactivities have been well characterized [5,11]. Cembranoid diterpenes, such as crassolide, have been found to have anti-inflammatory [8,12,13] and immunomodulatory activities [10,14]. Recent reports have indicated that crassolide exerts cytotoxic effects on A-549 (human lung adenocarcinoma), HT-29 (human colon adenocarcinoma), KB (human nasopharyngeal carcinoma), and P-388 (mouse lymphocytic leukemia) cells in culture [15,16]. However, while these studies show that crassolide is cytotoxic, the molecular mechanisms underlying the anticancer action of crassolide are unknown. As a result, the anticancer effects of crassolide on human lung adenocarcinoma cells were investigated in this study. The molecular processes underlying these anticancer effects were also examined.

## 2. Results

### 2.1. Crassolide Decreased Cell Viability and Colony Formation in Human Lung Cancer Cell Lines

**Figure 1:**
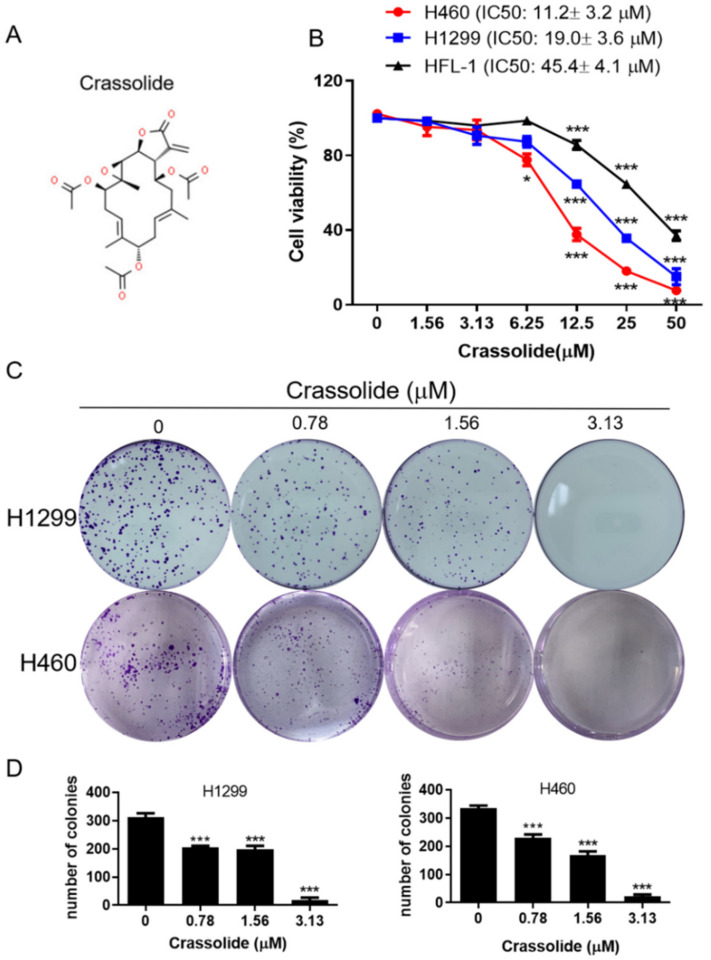
(**A**) Chemical structure of crassolide. (**B**) H460, H1299, and HFL-1 cells were treated with crassolide (0 to 50 μM) for 24 h. The cell viability was detected via an MTT assay. (**C**) Colony formation assays of H1299 and H460 cells treated with 0–3.13 μM crassolide for 7 days were demonstrated. (**D**) Data of colony formation assays are presented as mean ± SEM of three wells from one of three experiments. Compared to the DMSO-treated control group, significant differences are indicated by * *p* < 0.05, and *** *p*  <  0.001.

The chemical structure of crassolide is shown in Figure 1A. Treatment with crassolide (0–50 μM) for 24 h reduced the growth of human lung cancer cells (H1299 and H460) and normal human lung fibroblasts (HFL-1) in a dose-dependent manner, as shown in Figure 1B. The IC50 values of crassolide were 10.2 ± 3.5, 19.3 ± 4.1 μM, and 19.3 ± 4.1 M in the H460, H1299, and HFL-1 cells, respectively, according to the MTT test results. The results showed that the compound could decrease cell viability in a dose-dependent manner and was much more detrimental in cancer cells than in normal fibroblasts. Similarly, colony formation experiments revealed dose-dependent suppression of H1299 and H460 cell colony formation (Figure 1C,D) after 7 days of treatment with crassolide, confirming the growth-inhibitory effects of crassolide. In our study, H460 cells were more sensitive to crassolide than H1299 cells; thus, H460 cells were considered an adequate in vitro model for investigating the anticancer mechanism of crassolide.

### 2.2. Crassolide Causes G2/M Phase Arrest and Apoptosis in H460 Cells

**Figure 2:**
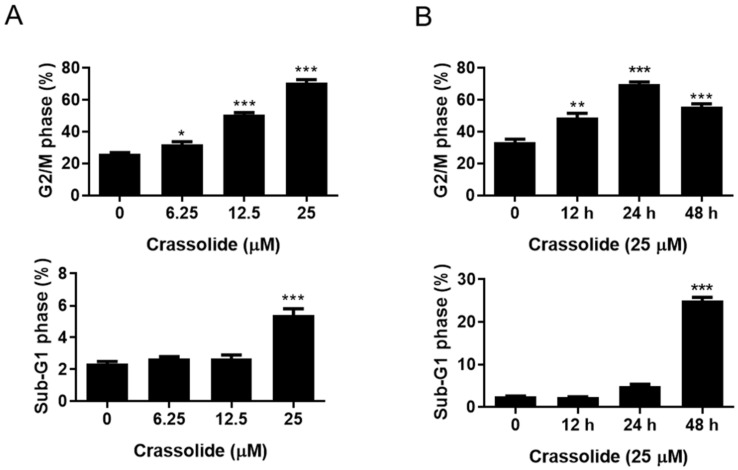
The effects of crassolide on cell cycle progression in H460 cells. The percentages of H460 cells in different cell cycle phases after treatment with different doses of crassolide for 24 h and treatment with 25 µM crassolide for different time points. (**A**) Percentages of H460 cells in the G2/M and sub-G1 phases after treatment with different concentrations of crassolide for 24 h. (**B**) Percentages of cells in the G2/M and SubG1 phases after incubation with 25 μM crassolide for different incubation times. Data are presented as mean ± SEM of three wells from one of three experiments. Compared to the DMSO-treated control group, significant differences are indicated by * *p* < 0.05, ** *p*  <  0.01, and *** *p*  <  0.001.

**Figure 3:**
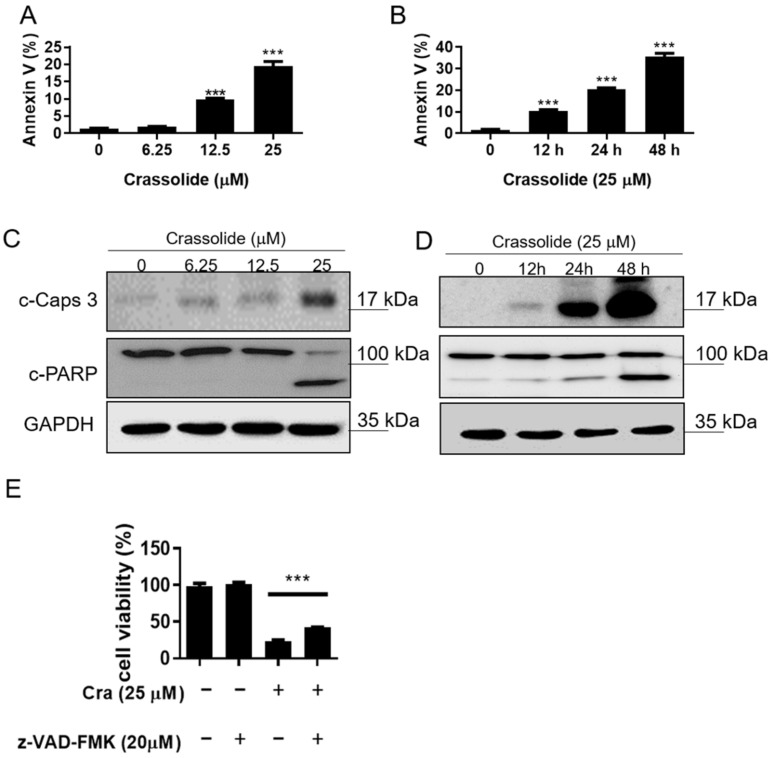
The effect of crassolide on caspase-dependent apoptosis of H460 cells. (**A**) H460 cells were treated with different concentrations of crassolide for 24 h or (**B**) subjected to treatment with 25 µM crassolide for different time points. We measured phosphatidylserine externalization and DNA integrity by FITC-annexin-V and PI, respectively. Annexin-V+/PI− staining (lower-right quadrant) indicates early apoptosis, while Annexin V+/PI+ staining (upper-right quadrant) represents late apoptosis. Data are presented as mean ± SEM of three wells from one of three experiments. Significant differences from the DMSO-treated control group were indicated by *** *p*  <  0.001. The expression of cleaved caspase-3 and PARP was measured by Western blotting after treatment with crassolide at different doses (**C**) or at different times (**D**). Data are presented as mean ± SEM (n = 3) for three independent experiments. (**E**) The viability of H460 cells after treatment with a pan-caspase inhibitor (Z-VAD-FMK) and crassolide. After 24 h, cell proliferation was measured using the MTT assay. Data are presented as mean ± SEM of three wells from one of three experiments, while *** *p*  <  0.001 indicates a significant difference from the group treated with crassolide alone.

H460 cells were treated with 0, 6.25, 12.5, and 25 μM crassolide for 24 h or with 25 μM crassolide for 0, 12, 24, and 48 h to determine whether crassolide induces cell death by cell cycle arrest and apoptosis. PI staining and flow cytometry were used to determine the DNA content of the cells. As shown in Figure 2A, treatment with 12.5 and 25 µM crassolide for 24 h increased the cell population in the G2/M phase compared to the control treatment (0.1% DMSO), with a corresponding reduction in the cell population in the G1 phase observed. In addition, 25 μM crassolide increased the number of cells in the sub-G1 phase, indicating dose-dependent apoptotic cell death (Figure 2A and Supplement Figure S1A). Moreover, there was a time-dependent increase in the number of cells arrested in the G2/M and sub-G1 phases (Figure 2B and Supplement Figure S1B). We further confirmed whether crassolide induces apoptosis using Annexin V-FITC and propidium iodide (PI) staining and flow cytometry analysis. As shown in Figure 3, treatment with crassolide induced a dose-dependent (Figure 3A and Supplement Figure S2A) and time-dependent (Figure 3B and Supplement Figure S2B) increase in the number of Annexin V^+^ apoptotic cells. We also measured the expression of cleaved caspase-3 and poly ADP-ribose polymerase (PARP) in H460 cells to examine whether the caspase-mediated pathway is involved in crassolide-induced apoptosis. As displayed in Figure 3C,D, cleaved caspase-3 and PARP expression in the crassolide-treated H460 cells increased in a dose- and time-dependent manner. Then, we utilized the MTT assay to assess the impact of a pan-caspase inhibitor (Z-VAD-FMK) on crassolide-initiated cell death. As shown in Figure 3E, pretreatment of H460 cells with Z-VAD-FMK significantly increased the viability of the cells treated with 25 μM crassolide for 24 h. Together, these outcomes suggest that crassolide inhibited cell viability through G2/M cell cycle arrest and promoted caspase-dependent apoptosis.

### 2.3. Crassolide Affected the Expression of G2/M Phase-Related Proteins in H460 Cells

**Figure 4:**
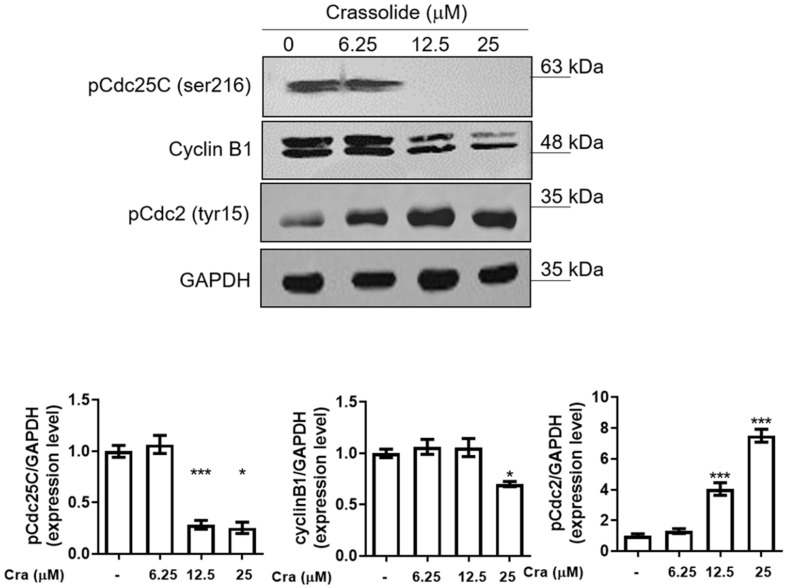
The effects of crassolide on G2/M cell cycle regulatory protein expression in H460 cells. H460 cells were treated with different concentrations of crassolide for 12 h, and Western blotting was performed to measure the expression levels of G2/M cell cycle regulatory proteins. GAPDH expression was used as an internal control to show equal protein loading. The bar graphs showed the quantified expression levels by ImageJ software. Data are presented as mean ± SEM (n = 3) for three independent experiments. Significant differences from the DMSO-treated control group are indicated by * *p* < 0.05, and *** *p*  <  0.001.

Next, we measured the expression of proteins related to the G2/M phase of the cell cycle after crassolide treatment. The results showed that phospho-Cdc25C (Ser-216) and cyclin B1 expression were reduced in a dose-dependent manner, but phospho-Cdc2 (Tyr-15) expression was increased at the same time point (Figure 4). These data indicated that crassolide could induce G2/M arrest by altering the expression of the corresponding G2/M phase-related proteins.

### 2.4. Crassolide-Triggered Autophagy in H460 Cells

**Figure 5:**
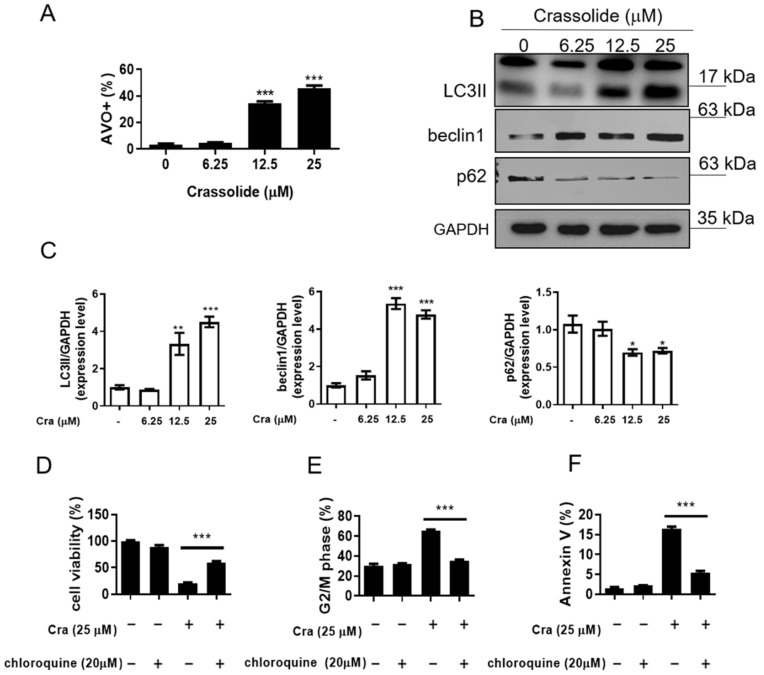
The effects of crassolide on autophagy in H460 cells. (**A**) The H460 cells were treated with different concentrations of crassolide for 24 h, and then, we harvested and stained them with acridine orange for assessment by flow cytometry. The mean ± SEM of experimental triplicates is presented in the bar graph. (**B**) The expression of the autophagy-related proteins LC3-II, beclin-1, and p62 is shown. The cells were treated with different concentrations of crassolide for 24 h, and protein expression was measured by Western blotting. GAPDH served as the internal control. (**C**) The mean ± SEM for three independent experiments is shown in the bar graph of Western blotting signal intensities; ImageJ quantified the data. Significant differences from the DMSO-treated control group are indicated by * *p* < 0.05, ** *p*  <  0.01, and *** *p*  <  0.001. The effect of chloroquine on the changes in cell viability, cell cycle progression, and apoptosis induced by crassolide. H460 cells were pretreated with 20 μM chloroquine (autophagy inhibitor) or control for 1 h before treatment with 25 μM crassolide for 24 h. (**D**) The cell viability was measured by MTT assay. (**E**) The cell cycle distribution and (**F**) the numbers of Annexin V+ apoptotic cells were analyzed by flow cytometry. Data are presented as mean ± SEM of three wells from one of three experiments, while *** *p*  <  0.001 indicates a significant difference from the group treated with crassolide alone.

The production of acidic vesicular organelles (AVOs), associated with autophagosomes and enhancing cell survival, was measured to assess autophagy, a lysosomal breakdown pathway [17]. To examine whether crassolide treatment affected the autophagy levels in H460 cells, we stained the cells with acridine orange and used flow cytometry to assess the autophagy levels. The results revealed that the cells exhibited significantly more AVOs after treatment with crassolide in a concentration-dependent manner (Figure 5A, Supplement Figure S3). In addition, Western blotting analysis revealed that the expression levels of the autophagy-related proteins LC3-II and beclin-1 were elevated, and p62 degradation was increased in crassolide-treated H460 cells (Figure 5B,C). Furthermore, we used chloroquine to inhibit autophagy and explored the effect of inducing G2/M arrest and apoptosis by crassolide. As indicated in Figure 5D, pretreatment with chloroquine significantly increased the viability of H460 cells. In addition, we observed that chloroquine significantly suppressed the crassolide-induced G2/M arrest (Figure 5E, Supplementary Figure S4A) and decreased the number of crassolide-induced Annexin V+ apoptotic cells (Figure 5F, Supplementary Figure S4B), as shown by flow cytometry. These data show that autophagy contributes to crassolide-induced G2/M arrest and apoptosis.

### 2.5. Crassolide-Activated Endoplasmic Reticulum (ER) Stress in H460 Cells

**Figure 6:**
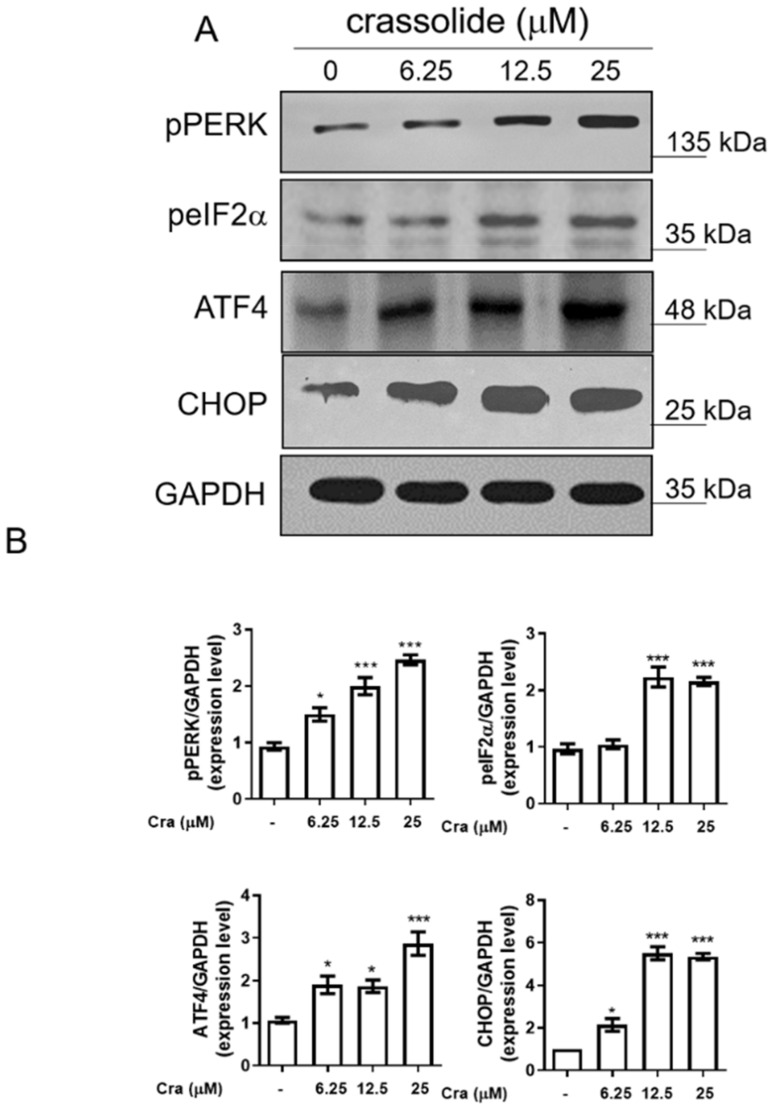
The effects of crassolide on ER stress pathway activation in H460 cells. (**A**) H460 cells were treated with different doses of crassolide for 12 h, and Western blotting was performed to measure the expression levels of ER stress regulatory proteins. GAPDH expression was used as an internal control to show equal protein loading. (**B**) The protein expression levels quantified by ImageJ software are shown in the bar graphs. Data are presented as mean ± SEM (n = 3) for three independent experiments. Significant differences from the DMSO-treated control group are indicated by * *p* < 0.05 and *** *p*  <  0.001.

**Figure 7:**
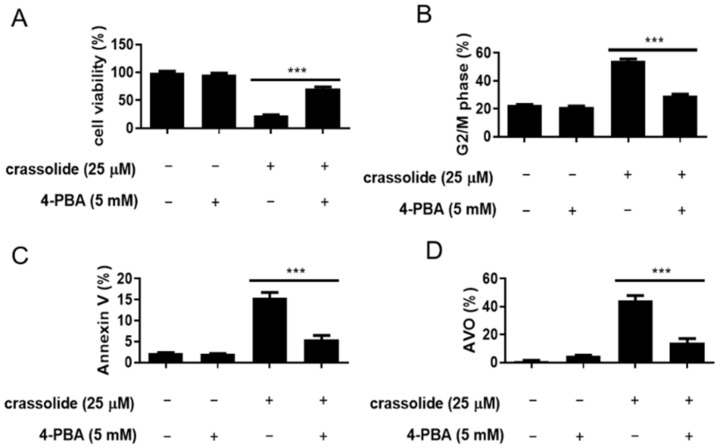
Effects of an ER stress inhibitor on the induction of cell death by crassolide in H460 cells. H460 cells were pretreated with the ER stress inhibitor 4-PBA (5 mM) or control for 1 h before treatment with 25 μM crassolide for 24 h. Then, (**A**) the cell viability was assessed by the MTT assay, (**B**) the cell cycle distribution, (**C**) the numbers of Annexin V+ apoptotic cells, and (**D**) autophagy (determined by acridine orange staining) were assessed by flow cytometry. All data are presented as the mean ± SEM of three wells from one of three experiments. Moreover, *** *p*  <  0.001 indicates a significant difference from the group treated with crassolide alone.

Prolonged ER stress can trigger G2/M arrest and cellular apoptosis [18,19]. We then investigated whether crassolide could activate ER stress to induce G2/M arrest and apoptosis. The expression of phospho-PERK, phospho-eukaryotic initiation factor-2α (p-eIF2α), ATF4, and CHOP, markers of ER stress, was investigated accordingly. To this end, we treated H460 cells with an increased dose of crassolide for 12 h. We found that the expression of these ER stress markers in the H460 cells was markedly increased by crassolide treatment (Figure 6A,B). In addition, a 2 h pretreatment with the ER stress inhibitor 4-phenylbutyric acid (4-PBA) suppressed the crassolide-mediated reduction in cell viability (Figure 7A), G2/M arrest (Figure 7B, Supplementary Figure S5A), apoptosis (Figure 7C, Supplementary Figure S5B), and AVO+ autophagy (Figure 7D, Supplementary Figure S5C) compared with treatment with crassolide alone, as shown by flow cytometry. Taken together, these data suggest that crassolide efficiently led to G2/M arrest, apoptosis, and autophagy by activating ER stress in the cells.

### 2.6. Crassolide-Induced Cell Growth Inhibition Was Associated with ROS Production

**Figure 8:**
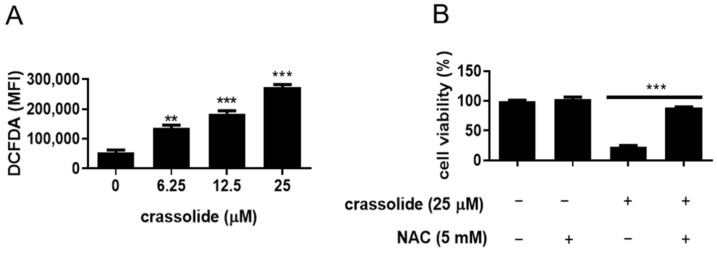
The effects of crassolide on ROS production in H460 cells. (**A**) H460 cells were treated with different concentrations of crassolide for 3 h, and the ROS levels were measured via flow cytometry. The mean ± SEM of the experimental triplicates is presented in the bar graph. Significant differences from the 0.1% DMSO-treated group are indicated by ** *p*  <  0.01, and *** *p*  <  0.001. (**B**) The effect of the ROS inhibitor NAC on the viability of crassolide-treated cells. H460 cells were pretreated with 5 mM NAC or control for 1 h before incubation with 25 μM crassolide for 24 h, and cell viability was determined by MTT assay. Data are presented as mean ± SEM of three wells from one of three experiments. Moreover, *** *p*  <  0.001 indicates a significant difference from the group treated with crassolide alone.

Since ROS can alter the cellular redox state and mitochondrial membrane potential, we further investigated whether crassolide-treated H460 cells produce ROS. As shown in Figure 8A and Supplement Figure S6, compared with that of DMSO-treated cells, the DCFDA fluorescence intensity of crassolide-treated cells was significantly increased in a dose-dependent manner. Moreover, a 2 h pretreatment with the oxidant scavenger N-acetylcysteine (NAC) suppressed the crassolide-mediated reduction in cell viability compared with treatment with crassolide alone (Figure 8B).

### 2.7. Crassolide Induced ROS-Mediated ER Stress Signaling and Upregulated G2/M Cell Cycle Arrest, Apoptosis, and Autophagy in H460 Cells

**Figure 9:**
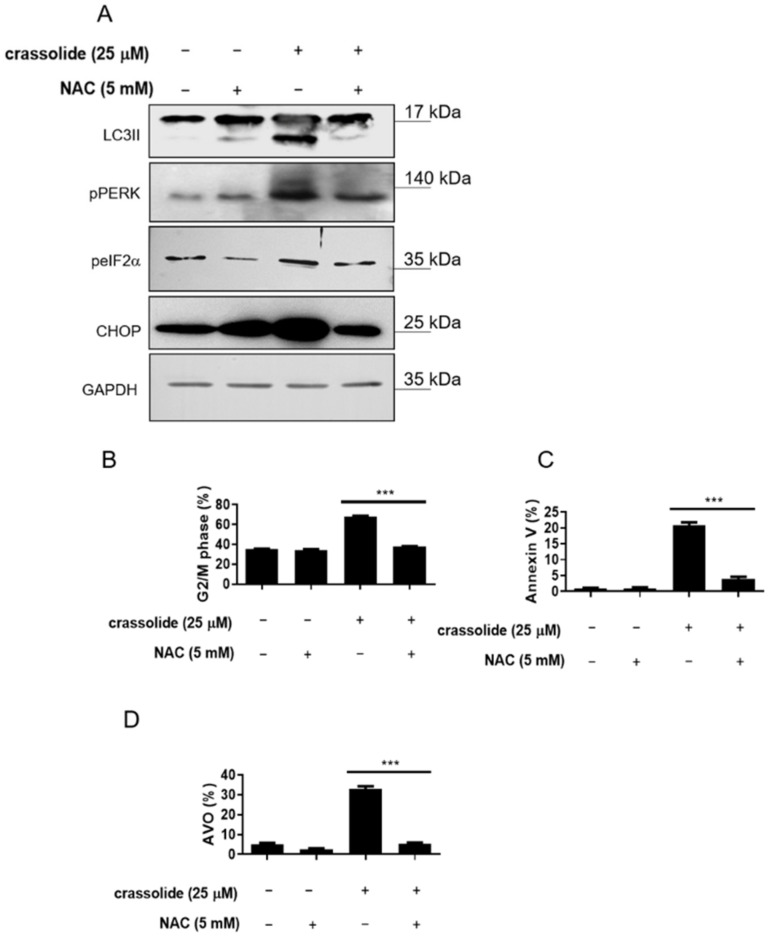
The effects of crassolide on the ROS-dependent activation of the ER stress pathway, G2/M cell cycle arrest, apoptosis, and autophagy in H460 cells. The H460 cells were pretreated with NAC for 1 h, treated with 25 μM crassolide for an additional 24 h, and then harvested. (**A**) The expression of the autophagy marker (LC3-II) and the ER stress-related molecules (pPERK, peIF2α, and CHOP) was examined by Western blotting analysis. GAPDH was used as an internal control. (**B**) Cell cycle distribution, (**C**) the numbers of Annexin V+ apoptotic cells, and (**D**) autophagy (as measured by acridine orange) were assessed by flow cytometry. Data are presented as mean ± SEM of three wells from one of three experiments. Moreover, *** *p*  <  0.001 indicates a significant difference from the group treated with crassolide alone.

Activation of proapoptotic pathways, including the ER stress-induced cancer cell apoptosis pathway, was reported after ROS generation. Therefore, we investigated whether crassolide-mediated ROS production activates the ER stress pathway in H460 cells. Figure 9A showed that a 2 h pretreatment with the ROS inhibitor NAC suppressed the crassolide-induced expression of the autophagy marker LC3-II and the ER stress-related molecules pPERK, peIF2α, and CHOP. In addition, we observed that NAC significantly inhibited the crassolide-induced G2/M arrest (Figure 9B and Supplementary Figure S7A), apoptosis (Figure 9C and Supplementary Figure S7B), and autophagy (Figure 9D and Supplementary Figure S7C). These results indicate that the G2/M arrest, apoptosis, and autophagy induced by crassolide are, at least in part, mediated by the ROS-dependent ER stress pathway.

## 3. Discussion

We have conducted a primary study on the effects of crassolide, a cembranoid diterpene isolated from the soft coral *Lobophytum crassum*, on human lung cancer cells. We found that crassolide significantly inhibited human H460 lung cancer cell growth. This beneficial effect was mediated by the induction of G2/M arrest, autophagy, and apoptosis, which were mediated through the ROS-dependent ER stress pathway.

Previous investigations have shown that crassolide has cytotoxic effects on cancer cells [15,16]. The mechanism has not yet been identified. Reduced functions of the G2-M and S cell cycle checkpoints may result in lung cancer [17]. Some studies have revealed that promoting G2/M arrest can be an important approach for anticancer treatment [20], where phosphorylation on serine 216 of Cdc25 is a critical G2/M checkpoint [21]. In the cell cycle, Cdc25c acts as an important regulator of the G2/M checkpoint and promotes mitoses. The cell division cycle protein 2 homolog (CDC2) gene encodes cyclin-dependent kinase 1 (Cdk1) protein, which is essential for G1/S and G2/M phase transitions. In human gastric cancer cells, suppressing the expression of Cdc25C would promote the cyclin B1/CDK1 complex and result in G2/M arrest [22]. Specifically, the dephosphorylation of Cdc25C activates the Cdc25 as a phosphatase, which subsequently dephosphorylates Cdc2 at residue tyrosine 15, leading to G2-M transition for mitosis [23]. We observed the enhanced phosphorylation of Cdc2 at tyr15 (Figure 4), supporting that crassolide induced G2/M arrest via modulating the inhibitory phosphorylation, particularly for CdC2. Consistently, the decreased expression of cyclin B1 indicated the suppression of cell cycle progression. However, we also found a substantial decrease in the protein level of phosphorylated Cdc25 as the addition of crassolide increased (Figure 4), which implied the activation of this phosphatase instead of inactivation. To this discrepancy, we infer that crassolide may manage the dephosphorylation of Cdc2 and the expression of cyclin B1 through alternative mechanisms, e.g., bypassing the canonical pathway or masking the phosphorylation sites of Cdc25 by overwhelming crassolide to prevent the phosphorylated Cdc25 being recognized by the commercial antibody. Nevertheless, our study proposes that crassolide as a potential anticancer drug could toxify NSCLC via modulating cell cycle arrest.

Drugs that promote the G2/M phase arrest and apoptosis of lung cancer cells may be expected to be helpful in lung cancer therapy [24]. Apoptosis is a type of programmed cell death examined in lung cancer cells [25,26]. Our study has confirmed that crassolide causes the apoptosis of lung cancer cells by Annexin V-FITC and PI staining and flow cytometry. Apoptosis occurs by two principal pathways: the intrinsic or mitochondrial pathway and the extrinsic or death receptor pathway [27]. Activation of the intrinsic or mitochondrial pathway releases cytochrome c and cleaves pro-caspase-3 and pro-PARP. The two pathways ultimately initiate the cleavage of caspase-3 and result in apoptosis. In our study, crassolide-treated H460 cells had increased caspase-3 and PARP activity, and treatment with a pan-caspase inhibitor decreased this activity.

Autophagy is a pathway that allows the degradation of cellular components via the interaction of autophagosomes with lysosomes; autophagy scavenges and eliminates misfolded or aggregated proteins, clears damaged organelles, and increases cell viability [28]. Beclin 1 plays a vital role in autophagy and interacts with either PI3k class III or BCL-2 [29]. LC3-II, created by the conjugation of LC3-I to phosphatidylethanolamine, is specific to autophagosomes and autolysosomes [30]. P62 is a marker of autophagic degradation. Autophagic proteolysis includes an increased transformation of LC3-I to LC3-II, increased acidic vesicular organelle (AVO) formation, and decreased p62/SQSTM1 formation [31]. Our research observed that crassolide promoted the formation of autophagosomes, increased the numbers of acidic vesicular organelles and the expression of LC3-II and beclin-1, and decreased the levels of p62.

However, autophagy plays different roles in cancer cells, promoting survival or increasing death. Autophagy might play a significant role in the suppression of carcinogenesis. Cancer cells resistant to anticancer treatment seldom undergo apoptosis but are sensitive to autophagic cell death [32]. On the other hand, autophagy may promote cancer carcinogenesis and recycle intracellular organelles damaged by chemotherapy or radiotherapy [33]. However, some reports have revealed that autophagy-related protein expression in lung cancer cells is associated with a poor prognosis [34,35]. Therefore, autophagy plays a dual role in cancer cells. In our research, the autophagy inhibitor chloroquine suppressed the ability of crassolide to induce G2/M arrest and apoptosis.

The ER is a fundamental intracellular organelle that functions in protein folding, translocation, and post-translational modification. When cancer cells are damaged by biochemical agents, oxidative stress, or DNA damage, ER stress will occur due to the accumulation of unfolded or misfolded proteins in the ER. Intrinsic adaptive mechanisms in cancer cells, such as misfolded protein accumulation during ER stress, improve cancer cell survival [36]. ER stress protein (XBP1s/GRP78) expression is also associated with a poor prognosis in lung cancer [37]. In contrast, persistent unresolved ER stress induces G2/M arrest and apoptosis [38,39]. Another study also demonstrated that persistent and severe ER stress could induce apoptosis, autophagy, necroptosis, or immunogenic cell death, and an induction of ER stress might become a strategy for anticancer treatment [40]. In our research, ER stress marker expression was increased in crassolide-treated cancer cells in a dose-dependent manner. Treatment with an ER stress inhibitor also decreased the occurrence of G2/M arrest, autophagy, and apoptosis.

ROS have a solitary unpaired electron in their outer electron shell, forming radicals, molecules, and ions [41]. Excessive ROS production can increase macromolecule oxidation, mtDNA mutation, aging, and cell death [40,42]. ROS accumulation mediates apoptosis [43]. Moreover, a protective autophagic response is also initiated in response to increasing intracellular ROS production [44]. ROS production is also associated with the apoptosis and G2/M arrest caused by anticancer agents [45]. ROS accumulation can cause cell injury and induce apoptosis, G2/M arrest, and autophagy. An essential inhibitor of ROS, NAC, was used to confirm the role of ROS in cell death. In our study, ROS were produced and accumulated in the crassolide-treated lung cancer cells. NAC reversed the ROS-mediated effects of crassolide, including G2/M arrest, apoptosis, LC3-II expression, and autophagy. Thus, ROS plays a vital role in the crassolide-mediated anticancer effect by triggering G2/M arrest, autophagy, and apoptosis.

In most mammalian cells, the unfolded protein response branches have been distinguished: the PERK-eIF2α-ATF4-CHOP pathway, the ATF6 pathway, and the IRE1α-XBP1 pathway [46]. The expression of three ER stress-related proteins, PKR-like ER kinase (PERK), inositol-requiring catalyst 1, and enacting record calculate 6, was increased during the crassolide-induced cellular breakdown of lung cells [47]. It was also shown that crassolide activates the ER stress pathway by increasing the protein levels of p-eIF2α and CHOP. Crassolide similarly activated ER stress to increase intracellular ROS levels in our study. To investigate the relationship between ROS and ER stress, we used NAC. The ROS-induced oxidative stress initiated by crassolide, as well as the ER stress and mitochondrial dysfunction, were recovered by NAC, which is a ROS inhibitor. One study also revealed a mechanism of anticancer effects via the ROS-dependent ER stress pathway [48,49].

**Figure 10:**
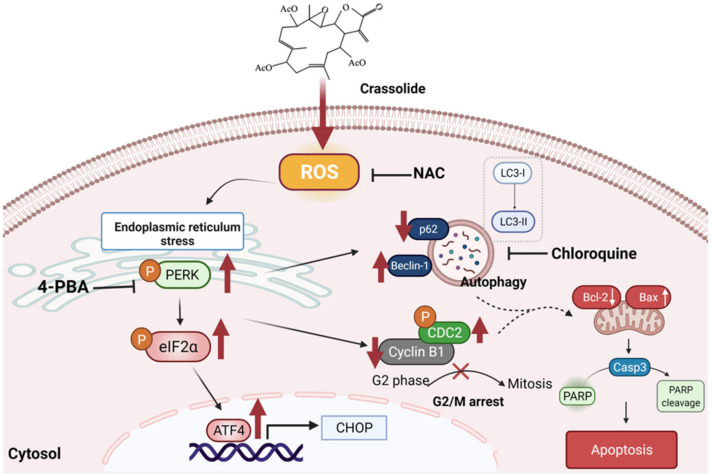
Our proposed model of the mechanism underlying the antitumor effects of crassolide on NSCLC cells. Crassolide induces autophagy-mediated cell death and G2/M arrest in human H460 NSCLC cells via ROS-mediated ER stress pathway activation.

A previous study found that some compounds induced lung cancer cell autophagy and apoptosis via the ROS and MAPK pathways [50,51]. ER stress and G2/M arrest were also reported to be induced by other compounds in lung cancer treatment [52,53]. Apoptosis and autophagy sometimes have synergetic effects. Apoptosis, autophagy, G2/M arrest, and ROS pathways could cooperate [54], which can be further investigated in lung cancer treatment. Taken together, these findings of our study demonstrate that crassolide promoted G2/M arrest, autophagy, and apoptosis in lung cancer cells via the ROS-dependent ER stress pathway. These phenomena are graphically summarized in Figure 10.

## 4. Material and Methods

### 4.1. Cell Culture

The H460 (BCRC Number: 60373) non-small-cell lung cancer cell line and HFL1 (BCRC Number: 60299) normal human lung fibroblast were purchased from the Food Industry Research and Development Institute (Hsinchu City, Taiwan). H1299 (ATCC Number: CRL-5803) was purchased from the American Type Culture Collection (Manassas, VA, USA). H460 and H1299 cell lines were maintained in RPMI-1640 medium, and HFL-1 was maintained in FK12 medium supplemented with 10% heat-inactive fetal bovine serum (FBS), 100 U/mL penicillin, and 100 g/mL streptomycin (#03-033-1B, Biological industries. Kibbutz, Israel). The cells were incubated at 37 °C in a humidified environment with a CO2/air ratio of 5%/95%.

### 4.2. Chemicals

Dr. Jui-Hsin Su (National Museum of Marine Biology and Aquarium, Pingtung, Taiwan) provided crassolide isolated from the wild-type soft coral *Lobophytum crissum* [14]. The stock solution was prepared in dimethyl sulfoxide (DMSO) at 20 mg/mL (Sigma-Aldrich, St. Louis, MO, USA). The working solutions were prepared by dilution with medium to the desired concentrations. Based on 1H-NMR and mass spectrum studies, the crassolide was 100% pure.

### 4.3. MTT Cell Viability Assay

The cells were seeded in 24-well plates at a density of 2 × 10^4^ cells per well and treated with crassolide at increasing doses or DMSO (0.1%) as vehicle control for 24 h. Then, each well received 300 μL of 3-(4,5-dimethylthiazol-2-yl)-2,5-diphenyltetrazolium bromide (MTT, Sigma-Aldrich, St. Louis, MO, USA) solution (0.5 mg/mL final concentration). The supernatant was aspirated after a 4 h incubation, and 600 μL of DMSO was added. A microplate reader was used to measure the absorbance at 570 nm (TECAN, Durham, NC, USA). The data are presented as the absorbance of crassolide-treated cells relative to DMSO-treated cells. GraphPad Prism software (Version 8.0, San Diego, CA, USA) was used to calculate the 50% inhibitory concentration (IC50) values.

### 4.4. Colony Formation Assays

The effect of crassolide on the clonogenicity of H1299 and H460 cells was investigated using colony formation assays. The cells were seeded in six-well plates at a density of 500 cells per well and incubated for 24 h. The cells were then treated with different concentrations of crassolide for 7 days to allow colonies to form. The colonies were stained with 2% crystal violet (Sigma-Aldrich, St. Louis, MO, USA), and the number of colonies in each well was counted under an inverted microscope (Olympus, Tokyo, Japan).

### 4.5. Determination of DNA Content by Flow Cytometry

Cells were seeded into 6-well plates (2 × 10^5^ cells/well) and treated with crassolide for 24 h. Trypsin was used to harvest the cells and then washed twice with PBS before being fixed with 70% ethanol overnight at 20 °C. The fixed cells were stained in PI solution comprising 1 mL of PBS, 50 g/mL PI (Sigma-Aldrich, St. Louis, MO, USA), 100 g/mL RNase A (Sigma-Aldrich, St. Louis, MO, USA), and 0.1% Triton X-100 (Sigma-Aldrich, St. Louis, MO, USA) in the dark at room temperature. An AccuriTM C5 cytometer (BD Biosciences, Franklin Lakes, NJ, USA) was used to detect the population of cells in each phase of the cell cycle, and the results were analyzed using BD Accuri C6 Software 1.0.264.21 (BD Biosciences, Franklin Lakes, NJ, USA).

### 4.6. Analysis of Cell Apoptosis by Flow Cytometry

The degree of apoptosis was assessed with an Annexin V-FITC/PI apoptosis detection kit (Biolegend, San Diego, CA, USA). A total of 2 × 10^5^ cells/well were treated with crassolide for 24 h before being harvested and washed three times with PBS. The cells were incubated for ten minutes in the dark with 5 μL Annexin V-FITC (20 μg/mL) and 10 μL PI. The apoptotic cells were detected using a BD Accuri C5 cytometer, and the results were analyzed using BD Accuri C6 Software 1.0.264.21.

### 4.7. Western Blotting Analysis

A total of 2 × 10^5^ cells/well were seeded in 6-well plates and treated with crassolide for the indicated time. The cells were lysed with RIPA buffer (Sigma-Aldrich, St. Louis, MO, USA) supplemented with a 1% protease inhibitor cocktail and 2% phenylmethanesulfonylfluoride fluoride (PMSF; Sigma-Aldrich, St. Louis, MO, USA). The BCA Protein Assay Kit (Visual Protein, Taipei City, Taiwan) was used to measure the protein concentration. A 12% SDS–PAGE gel was used to separate the cell lysates, and the proteins were electroblotted onto Immobilon-P Transfer Membranes (Merck Millipore, Burlington, MA, USA). The membranes were hybridized with anti-cleaved caspase-3 (Asp175) (#9661, Cell Signaling, Danvers, MA, USA), anti-cleaved PARP (#9542, Cell Signaling, Danvers, MA, USA), anti-pCDC25c (Ser216) (clone 63F9) (#4901s, Cell Signaling, Danvers, MA, USA), anti-Cyclin B1 (clone GNS1) (Santa cruz, CA, USA), anti-pCDC2 (Tyr 15) (#A1256-100, BioVision, Milpitas, CA, USA), anti-LC3-II (#AB51520, Abcam, Waltham, MA, USA), anti-Beclin1 (clone D40C5) (#3495S, Cell Signaling, Danvers, MA, USA), anti-p62 (clone H-290) (#sc-25575, Santa Cruz, CA, USA), anti-phospho-PERK (Thr 980)(#bs-3330R, Bioss inc, Woburn, MA), anti-phospho-eIF2α (119A11) (# 3597s, Cell Signaling, Danvers, MA, USA), anti-ATF-4 (clone D4B8) (#11815s, Cell Signaling, Danvers, MA, USA), anti-CHOP (clone L63F7) (#2895, Cell Signaling, Danvers, MA, USA) and anti-GAPDH (clone 6C5) (#ab8245, Abcam, Cambridge, MA, USA) antibodies. The membranes were then incubated with suitable HRP-conjugated secondary antibodies (Jackson ImmunoResearch Laboratories, West Grove, PA, USA) overnight at 4 °C. The enhanced chemiluminescence detection kit reagent was used to detect the bands (Millipore, Burlington, MA, USA), and the bands were imaged using a Hansor Luminescence Image System (Taichung, Taiwan). The GAPDH level in each lane was used to normalize all the bands. The NIH ImageJ 1.47 application for Windows (Bethesda, MD, USA) was used to measure the intensity of the bands.

### 4.8. Flow Cytometric Analysis of ROS Levels

A total of 2 × 10^5^ H460 cells were seeded in a six-well plate. The cells were then treated with crassolide or DMSO. After 24 h of treatment, the cells were centrifuged, washed with PBS, and then stained with 1 l of 5-(and-6)-carboxy-2′,7′-dichlorodihydrofluorescein diacetate (Sigma-Aldrich, St. Louis, MO, USA) for 10 min at room temperature in the dark. Then, the cells were analyzed in the FL-1 channel of an Accuri C5 cytometer.

### 4.9. Flow Cytometric Analysis of Acridine Orange Levels

H460 cells were seeded at a density of 2 × 10^5^ cells/well in a six-well plate. The next day, the cells were treated with crassolide or 0.4% DMSO for 24 h. The cells were then incubated with acridine orange (1 μg/mL) (Sigma-Aldrich) for 15 min at 37 °C. After removing the solution, the wells were filled with PBS, and the cells were examined on an Accuri 5 flow cytometer with C6 Accuri system software.

### 4.10. Inhibitor Analysis

To examine whether caspases, autophagy, ER stress, and ROS participated in the crassolide-induced reduction in cell viability, H460 cells were grown overnight and then pretreated with a pan-caspase inhibitor (Z-VAD-FMK) (#V116, Sigma-Aldrich, St. Louis, MO, USA), an autophagy inhibitor (chloroquine) (#14194, Cayman, MI, USA), an ER stress inhibitor 4-PBA (#11323, Cayman, MI, USA), or a ROS inhibitor (NAC) (#A7250, Sigma-Aldrich, St. Louis, MO, USA) for 2 h. The cells were then treated with 25 μM crassolide for 24 h and subjected to cell viability, apoptosis, cell cycle, and autophagy correlation analyses.

### 4.11. Statistical Analysis

One-way ANOVA was used to compare the data from several groups (GraphPad Prism Version 8.0) (San Diego, CA, USA). A probability (*p*) value of 0.05 was used to determine statistical significance.

## 5. Conclusions

In summary, the anti-proliferative effect of crassolide and its underlying mechanism were studied in the H460 NSCLC cell line. We found that apoptosis-related proteins, autophagy-related proteins, and G2/M-related proteins were expressed at higher levels after crassolide treatment in vitro, and these effects mediated ER stress activation by ROS accumulation. Further studies will be performed in vivo to confirm these effects of crassolide. We hope our findings will allow crassolide to be developed for clinical use in the future.

## Data Availability

The data that support the findings of this study are available from the corresponding author upon reasonable request.

## Supplementary Materials

The following supporting information can be downloaded at: https://www.mdpi.com/article/10.3390/ijms23105624/s1.
