# Supplementary material for: Crassolide Induces G2/M Cell Cycle Arrest, Apoptosis, and Autophagy in Human Lung Cancer Cells via ROS-Mediated ER Stress Pathways
Source: Int J Mol Sci. 2022 May 17;23(10):5624. doi: 10.3390/ijms23105624 (PMC9144222; doi:10.3390/ijms23105624)
Supplement: Supplementary file 1 [file ijms-23-05624-s001.zip › ijms-1651524-supplementary.pdf]

Supplement Figures

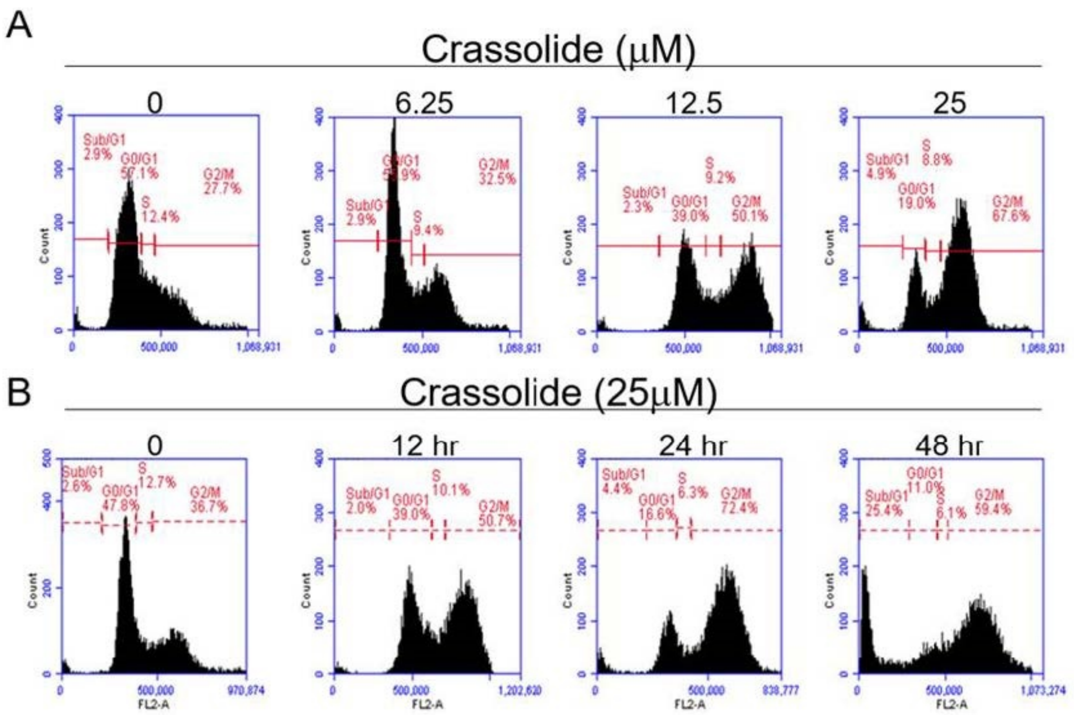

Supplement Figure S1. The effects of crassolide on cell cycle progression in H460 cells. (A) The percentages of H460 cells in different cell cycle phases after treatment with different doses of crassolide for 24 hours and (B) treatment with 25 μM crassolide for different time points.

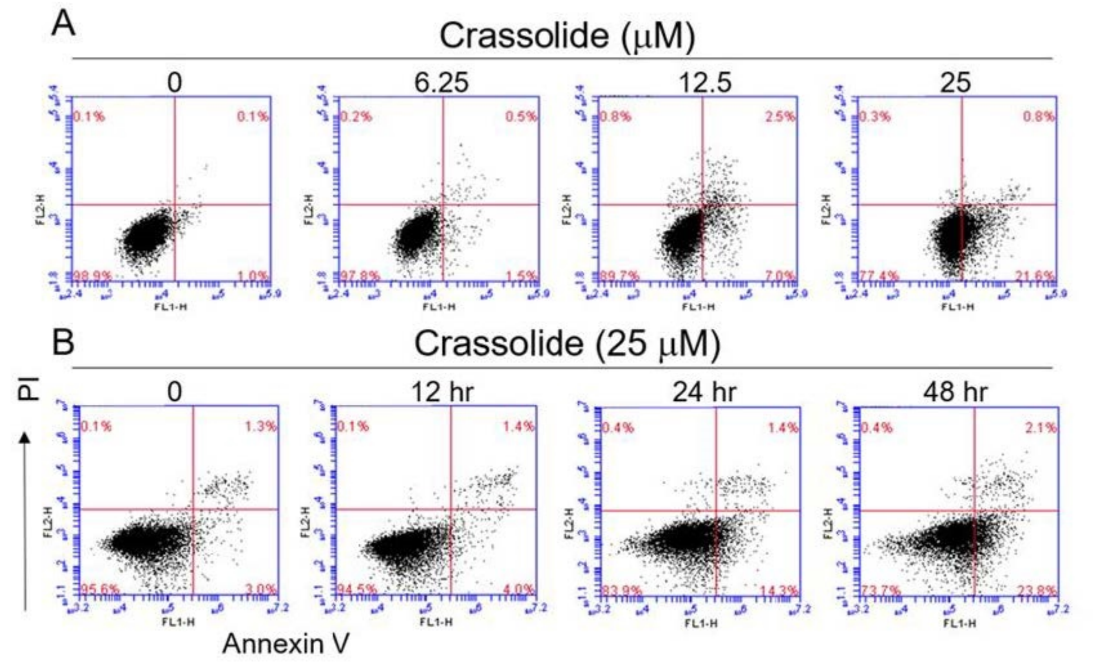

Supplement Fig. S2. The effect of crassolide on caspase-dependent apoptosis of H460 cells. (A) H460 cells were treated with different concentrations of crassolide for 24 hours or (B) subjected to treatment with 25 μM crassolide for different time points.

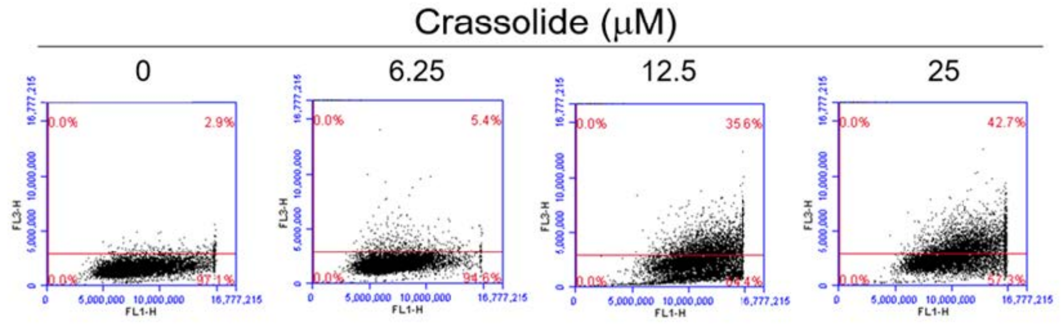

Supplement Figure S3. The effects of crassolide on autophagy in H460 cells. The H460 cells were treated with different concentrations of crassolide for 24 hours, and then we harvested and stained them with acridine orange for assessment by flow cytometry.

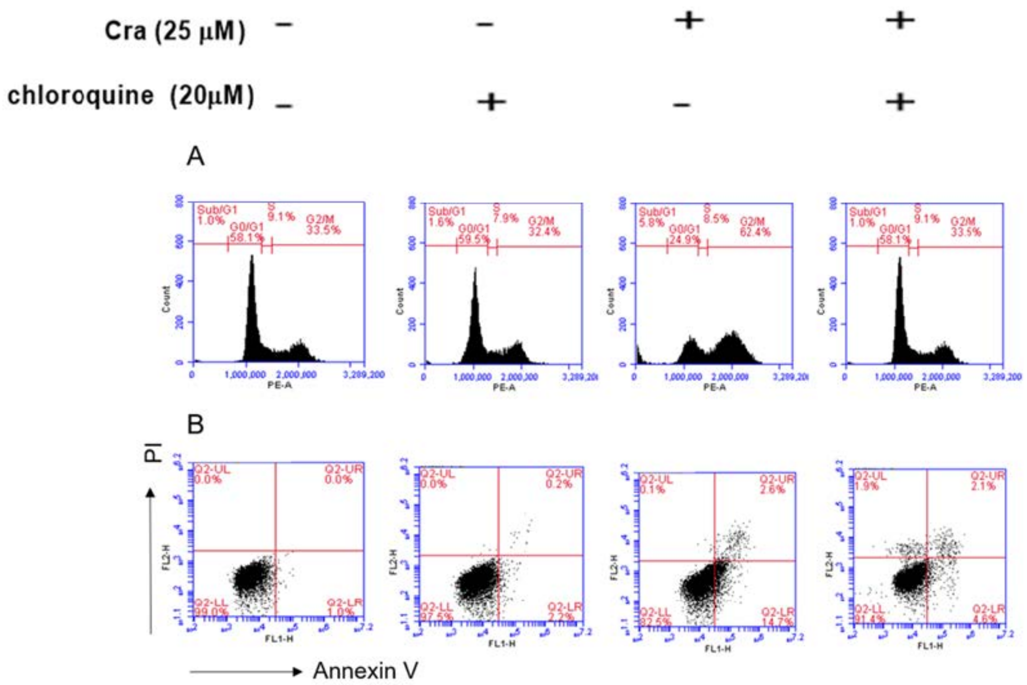

Supplement Figure S4 The effects of the autophagy inhibitor chloroquine on cell cycle arrest and apoptosis induced by crassolide in H460 cells. (A) Cell cycle distribution and (B) numbers of Annexin V+ apoptotic cells were determined by flow cytometry.

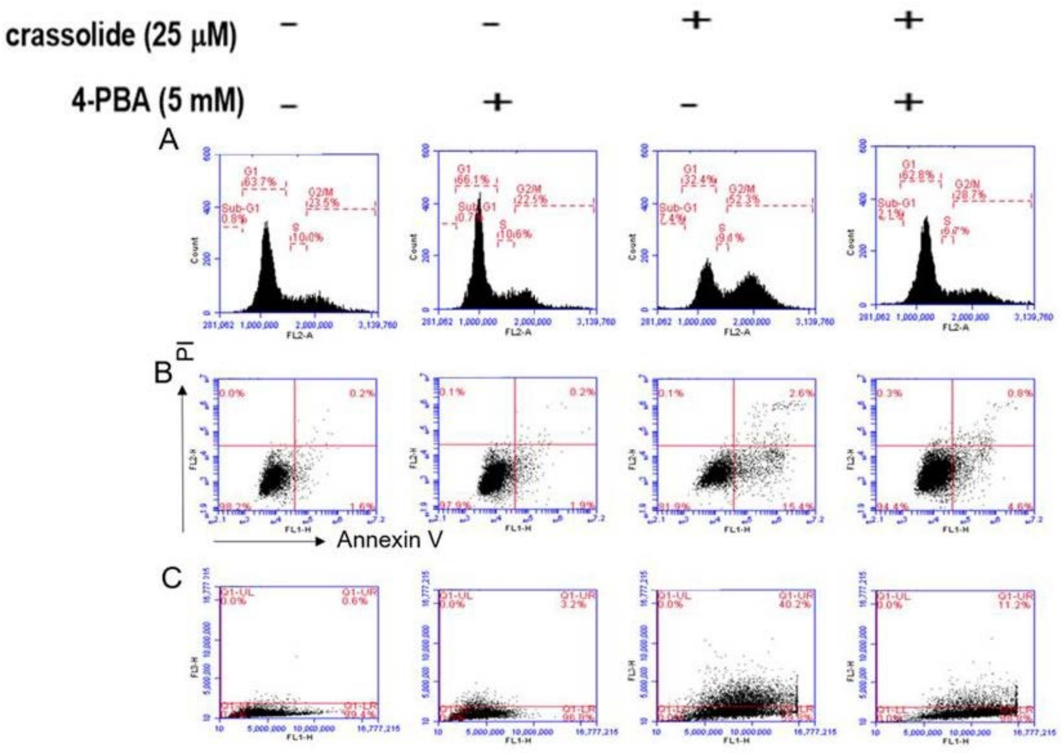

Supplemental Figure S5 The effects of the ER stress inhibitor 4-PBA on the cell cycle arrest, apoptosis, and acidic vesicular organelle formation induced by crassolide in H460 cells. (A) Cell cycle distribution, (B) numbers of Annexin V+ apoptotic cells, and (C) numbers of acridine orange-positive cells were assessed by flow cytometry.

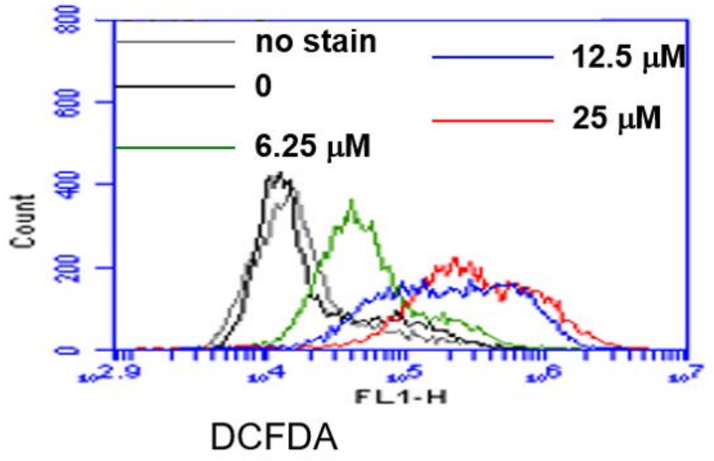

Supplemental Figure S6. The effects of crassolide on ROS production in H460 cells. H460 cells were treated with different concentrations of crassolide for 3 hours, and the ROS levels were measured via flow cytometry.

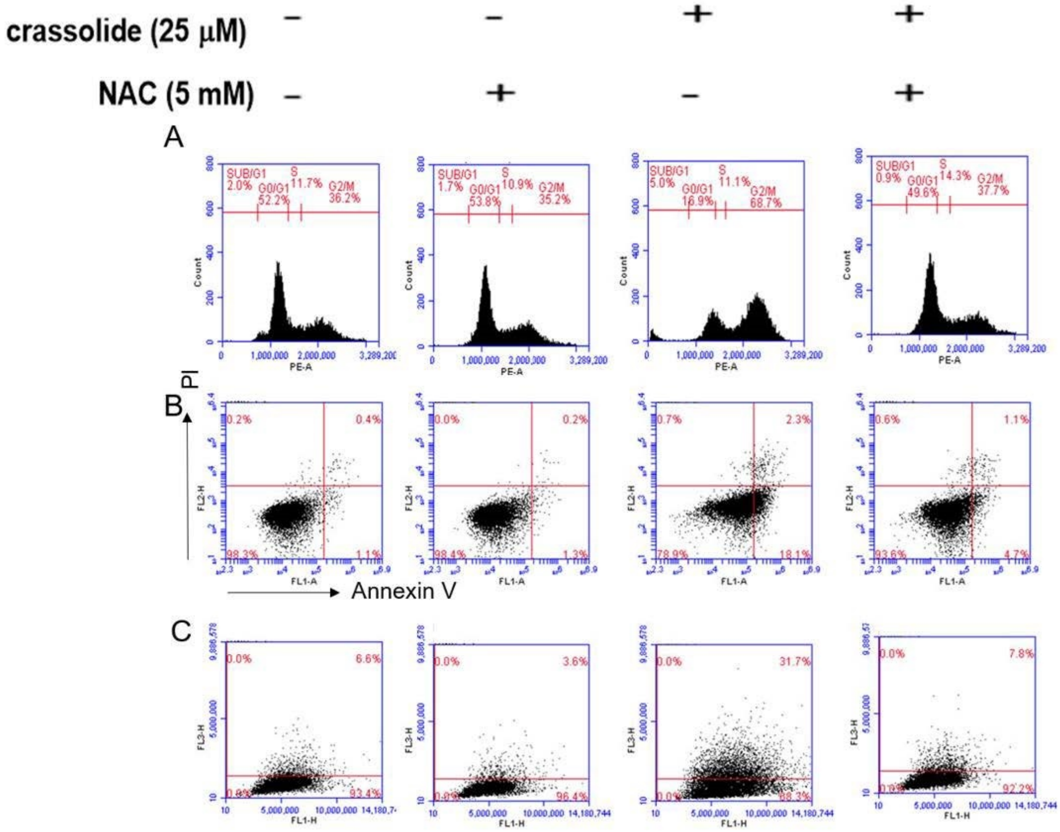

Supplement Figure S7 The effects of the ROS inhibitor NAC on the cell cycle arrest, apoptosis, and acidic vesicular organelle formation induced by crassolide in H460 cells. (A) Cell cycle distribution, (B) numbers of Annexin V+ apoptotic cells, and (C) numbers of acridine orange-positive cells were assessed by flow cytometry.
